# Gene expression analysis in EBV-infected ataxia-telangiectasia cell lines by RNA-sequencing reveals protein synthesis defect and immune abnormalities

**DOI:** 10.1186/s13023-021-01904-3

**Published:** 2021-06-28

**Authors:** Moussab Tatfi, Emeline Perthame, Kenzo-Hugo Hillion, Marie-Agnès Dillies, Hervé Menager, Olivier Hermine, Felipe Suarez

**Affiliations:** 1 INSERM U1163/CNRS ERL8254 - Laboratory of Cellular and Molecular Mechanisms of Hematological Disorders and Therapeutic Implications, Institut Imagine, Paris, France; 2 Department of Adult Hematology, AP-HP. Centre, Necker – Enfants Malades Hospital, Université de Paris, Paris, France; 3 Université de Paris, Paris, France; 4 Hub de Bioinformatique et Biostatistique – Département Biologie Computationnelle, Institut Pasteur, Paris, France

**Keywords:** EBV, ATM, Ataxia, RNA-seq, Lymphoma

## Abstract

**Background:**

Epstein–Barr virus (EBV) targets B-cells where it establishes a latent infection. EBV can transform B-cells in vitro and is recognized as an oncogenic virus, especially in the setting of immune compromise. Indeed, immunodeficient patients may fail to control chronic EBV infection, leading to the development EBV-driven lymphoid malignancies. Ataxia telangiectasia (AT) is a primary immune deficiency caused by mutations in the *ATM* gene, involved in the repair of double-strand breaks. Patients with AT are at high risk of developing cancers, mostly B-cell lymphoid malignancies, most of which being EBV-related. Aside from immune deficiency secondary to AT, loss of ATM function could also hinder the control of the virus within B-cells, favoring lymphomagenesis in AT patients.

**Results:**

We used RNA sequencing on lymphoblastoid cell lines derived from patients with AT and healthy donors to analyze and compare both cellular and viral gene expression. We found numerous deregulated signaling pathways involving transcription, translation, oncogenesis and immune regulation. Specifically, the translational defect was confirmed in vitro, suggesting that the pathogenesis of AT may also involve a ribosomal defect. Concomitant analysis of viral gene expression did not reveal significant differential gene expression, however, analysis of EBV interactome suggests that the viral latency genes EBNA-3A, EBNA-3C and LMP1 may be disrupted in LCL from AT patients.

**Conclusion:**

Our data support the notion that ATM deficiency deregulates cellular gene expression possibly disrupting interactions with EBV latent genes, promoting the oncogenic potential of the virus. These preliminary findings provide a new step towards the understanding of EBV regulation and of AT pathogenesis.

**Supplementary Information:**

The online version contains supplementary material available at 10.1186/s13023-021-01904-3.

## Background

Epstein–Barr virus (EBV) is a human *Herpesviridae* that infects about 95% of adults worldwide. Most genes encoded by the viral genome are expressed during the lytic cycle and contribute to the production of viral particles. By contrast, only a restricted repertoire of viral genes is expressed during latency, to allow a lifelong persistence of the virus in the organism. EBV’s lytic cycle takes place in the oropharyngeal epithelium whereas the latent cycle is established in the B lymphocytes from the underlying lymphoid tissues [1]. Chronic infection in immunocompetent individuals is generally asymptomatic with the virus being maintained in a latent state [2]. However, inefficient control of viral latency contributes to the development of malignancies such as Burkitt's lymphoma, Hodgkin’s lymphoma and Nasopharyngeal carcinoma.

Several primary immune deficiencies (PID) are associated with poor EBV responses and are also at high risk for EBV-related malignancies [3]. Ataxia telangiectasia (AT) is a rare PID caused by mutations in the *Ataxia Telangiectasia Mutated* (*ATM)* gene, involved in the DNA damage response (DDR). AT patients have an increased risk of cancer, mostly B-cell lymphoid malignancies, many of which are related to EBV [4]. The prevailing hypothesis to explain the increased incidence of malignancies in patients with AT is based on the role of the ATM kinase in the DDR [5]. However, the strong association of lymphomas with EBV also suggests an oncogenic role of the latter. AT patients often present with antibody deficiency and T-cell lymphopenia but rarely overt immunodeficiency [6]. A number of PIDs exhibit a selective susceptibility to EBV-related malignancies, while displaying a more restricted susceptibility to other opportunistic infections. In such cases, specific mechanisms may include pathways important for T, NK and iNKT cytotoxicity aimed at EBV-infected B-cells, and pathways involved in expansion of EBV-specific T-cells, leading to an inability to cope with intense EBV induced proliferative stress like in XMEN or CTPS1 mutated patients [7]. We raised the hypothesis that the lack of ATM function in AT patients may be associated with a less stringent control of EBV latency in ATM-deficient B cells, thereby promoting the oncogenic properties of the virus.

Indeed, beside DNA repair, ATM is also involved in a multitude of signaling pathways such as cell cycle checkpoint, apoptosis, mitochondrial metabolism and telomere maintenance [8]. In addition, ATM is involved both in transcription induction [9], and in transcription inhibition in the vicinity of a double-strand break, in nuclear [10] or ribosomal DNA [11]. ATM has a role in the control of the latent cycle of Kaposi’s sarcoma herpesvirus (KSHV) and Murine γ-herpesvirus 68 (MHV68), both related to EBV [12, 13].

We performed RNA sequencing (RNA-seq) on lymphoblastoid cell lines (LCL) generated from AT patients and healthy donors, to explore the specific expression pattern of both the cellular and viral genomes and investigate a possible role of ATM in the regulation of EBV latent cycle.

## Results

### Identification of differentially expressed genes

**Fig. 1:**
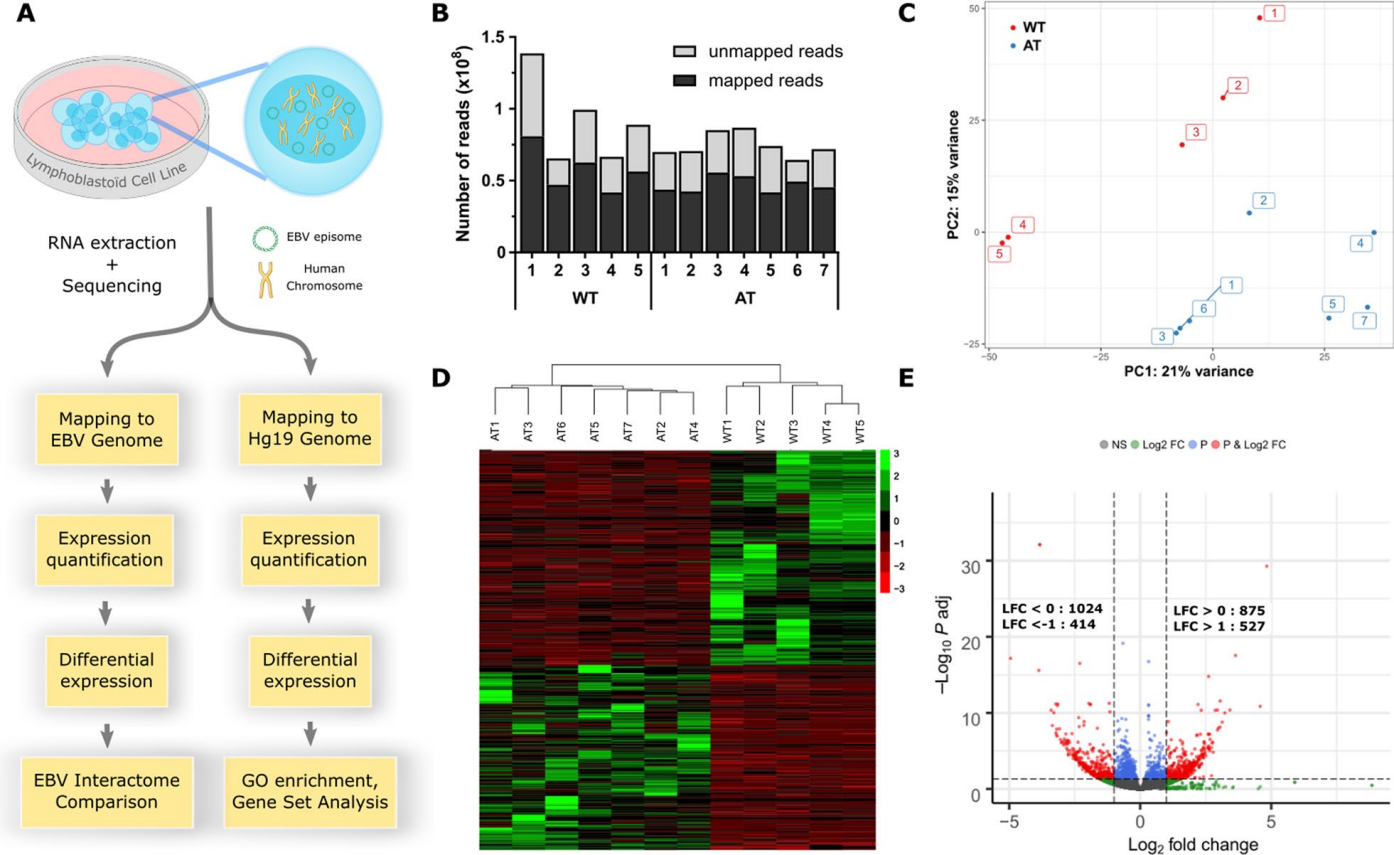
Overview of RNA-seq data generated from lymphoblastoid cell lines derived from 7 AT and 5 unaffected control individuals. **a** Experimental design and bioinformatic data analysis for dual RNA-seq. **b** Result of reads alignment on the human and EBV references genomes. For each sample, the number of mapped or unmapped read are shown on the y-axis. **c** Principal component analysis (PCA) plot visualizing the similarities between biological replicates and the separation between LCL-WT (red dots) and LCL-AT (blue dots). **d** Hierarchical clustering dendrogram of LCL-WT and LCL-AT normalized gene expression, with expression levels of differentially expressed genes with |log2FC|> 1 showed as a heatmap. Red represent downregulated genes and green represent upregulated genes. **e** Volcano plot displaying log2 fold change against − log10 (p-adjusted). DESeq2 analysis identified 1024 significant downregulated genes and 875 significant upregulated genes among which 414 had a log2 fold change < − 1 and 527 had a log2 fold change > 1

We conducted RNA-seq on LCL from AT patients (LCL-AT, n = 7) and healthy donors (LCL-WT, n = 5), previously phenotyped for ATM function (Additional file 1: Fig. S1), using Illumina HiSeq 2500 technology (Fig. 1a). An average of 82 million paired-end reads were generated per sequenced sample (range 64–138 million). Mapping on the human genome (GRCh38) and the viral genome (V01555.2) gave an average of 63% (range 56–76%) and 0.19% (range 0.10–0.44%) of mapped reads respectively (Fig. 1b). A total of 30,794 transcripts were detected using a threshold of one transcript in at least one sample, including 30,687 on the human genome and 107 on the viral genome. Principal component analysis (PCA) shows that LCL-AT and WT segregate into two distinct groups (Fig. 1c).

1899 (6.2%) of the genes were differentially expressed (adjusted *p* value < 0.05), among which 941 (49.5%) had an absolute log2 fold-change (|log2FC|) > 1. These genes and their corresponding fold-changes are given in Additional file 2: Table S1. A heatmap of the DE genes with |log2FC|> 1 demonstrating a separation of the 2 groups is shown in Fig. 1d. Of the 1899 DE genes, 875 were upregulated and 1024 were downregulated in AT (Fig. 1e).

### Analysis of the cellular genome

**Fig. 2:**
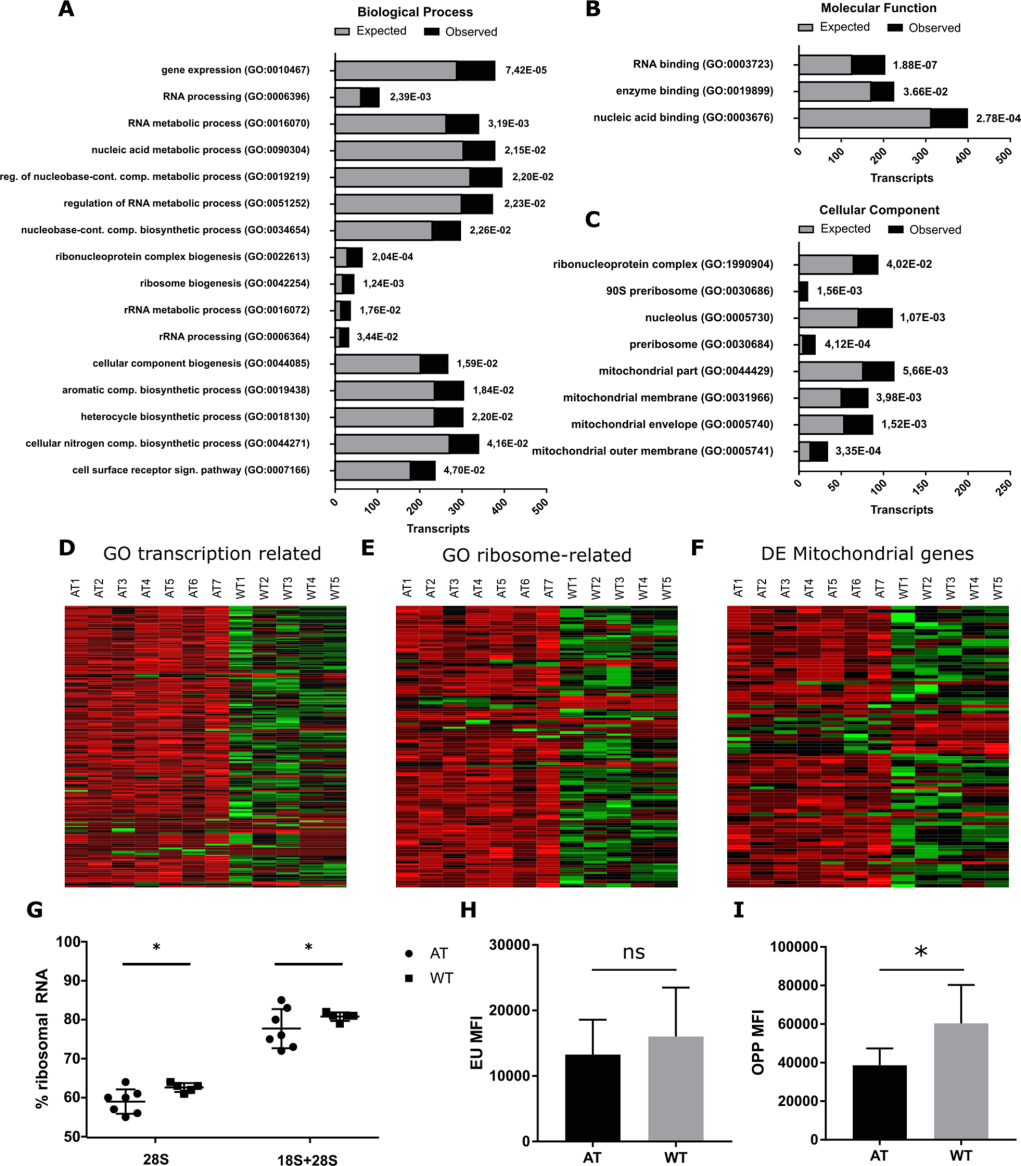
GO analysis of differentially expressed genes and functional exploration. **a**–**c** The PANTHER statistical over-representation test tool was used to determine over-representation of defined functional GO classes for the 1895 differentially expressed genes: **a** biological process; **b** molecular function; **c** cellular component. Expected and observed frequencies for each GO category are shown by gray and black bars respectively. The number of genes assigned to each category can be determined from the x-axis, and *p* value for each GO category is given on the right of each bar. **d** and **e** Non-redundant genes in over-represented GO categories were regrouped into functional classes and displayed as heatmaps: **d** GO categories related to transcription (GO:0010467; GO:0006396; GO:0016070; GO:0051252; GO:0003723; GO:0090304; GO:0019219; GO:0034654). **e** GO categories related to ribosome (GO:0022613; GO:0042254; GO:0016072; GO:0006364; GO:0030684; GO:0005730; GO:0030686; GO:1990904). **f** All mitochondrial differentially expressed genes, as determined by the BioMart R annotation package are displayed as a heatmap. **g** Expression of rRNA in LCL-AT and LCL-WT shown as a percentage of total RNA expression for 28S and 18S + 28S. **h** Analyze of nascent RNA transcription in LCL-AT and LCL-WT 1 h after 10 µM 5-ethynyl uridine (EU) incorporation. **i** Analyze of nascent protein synthesis in LCL-AT and LCL-WT 30 min after 50 µM O-Propargyl-puromycin (OPP) incorporation. **h** and **i** MFI: Mean fluorescence intensity. *pval < 0.05. Data are mean ± SD

We applied the over-representation tool from the PANTHER classification system to the list of DE genes. 1703 (90%) DE genes were annotated in the GO-database [14]. Over-represented categories, which are more highly represented in the DE gene list than would be expected by chance [15], were determined for biological process, molecular function and cellular component. This resulted in a total of 38 over-represented GO-categories (Fig. 2a–c). 11 GO categories corresponding to cellular component were judged as too general and non-informative (Additional file 3: Fig S2).

Interestingly, 8 GO-categories (21%) corresponding to general transcription, and 8 GO-categories (21%) corresponding to rRNA synthesis were over-represented in the DE genes list. The mitochondrial part was also over-represented with 4 GO-categories (11%). We isolated the gene list pertaining to the GO-categories related to general transcription, to rRNA synthesis and, using annotations from BioMart tool, all mitochondrial DE genes, including nuclear and mitochondrial encoded genes. A heatmap for each of these gene-sets was generated (Fig. 2d–f) and shows that LCL-AT have a defect in ribosome and transcription-related gene expression, and in mitochondrial genes. Gene lists related to each heatmap are given in Additional file 4: Table S2.

The rRNA fraction of the RNA prepared prior to RNA-seq was analyzed by capillary gel electrophoresis. The results show that LCL-AT have a slightly lower percentage of rRNA than LCL-WT (Additional file 5: Fig. S3A). Among these rRNAs, we observed a significant decrease in the percentage of the 28S RNA and 28S + 18S RNA (Fig. 2g) but no difference for the 18S RNA (Additional file 5: Fig. S3B). The 28S/18S ratio and the RNA integrity number (RIN) attested that RNA was of good quality and that there was no difference in RNA degradation between LCL-AT and LCL-WT (Additional file 5: Fig. S3C–D).

Transcription and translation rates were assessed by incubating LCL-WT and LCL-AT with 5-ethynyl uridine (EU), an RNA nucleotide analogue, or with O-Propargyl-puromycin (OPP), an agent incorporated during translation. EU incorporation did not show any significant difference in the transcription rate between LCL-WT and LCL-AT (Fig. 2h) but OPP incorporation showed a significant decrease (*p* value < 0.05) in protein synthesis rate in LCL-AT (Fig. 2i). These results indicated that LCL-AT have a generalized protein synthesis defect. Mitochondrial respiration was also assessed by SeaHorse analysis but did not show any difference between LCL-WT and LCL-AT (data not shown).

**Fig. 3:**
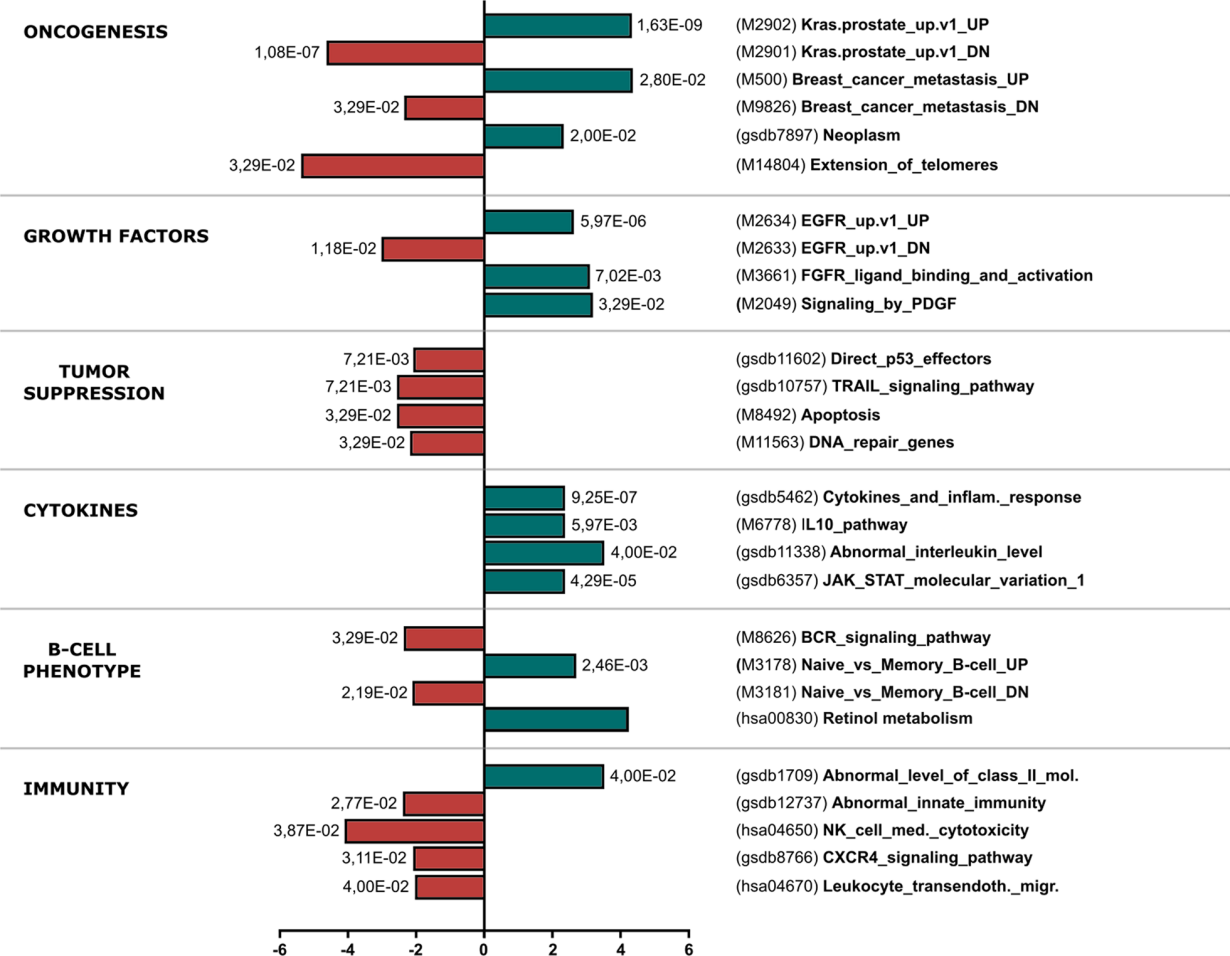
EGSEA analysis of differentially expressed genes. The EGSEA algorithm was used to identify significantly induced or inhibited Gene-Sets from the MSigDB, GSetDB and KEGG databases. Significantly induced (green) or inhibited (red) Gene-Sets of interest are displayed, as given by EGSEA results with |avg.logfc.dir|> 2 and *P*.adj < 0.05. Interesting Gene Sets were reordered in functionnal categories: Oncogenesis, Growth Factors receptors, Tumor suppression, Cytokines, B-cell phenotype and Immunity. The avg.logfc.dir value is indicated on the x-axis and the *P*.adj on the top of each bar

In order to further analyze the DE gene pathways, we performed the Ensemble of Gene Set Enrichment Analyses (EGSEA) [16]. Given the large amount of data generated (Additional file 6: Table S3), we kept only Gene-Sets (GS) with adjusted *p* value < 0.05 and absolute average log2FC with genes regulated in the same direction as the GS (|avg.logfc.dir|) > 2 (Fig. 3).

GS related to cancer were particularly enriched. DE genes were enriched in the signature of the KRAS-dependent Prostate Cancer in both directions. This trend was also corroborated by the GS “Neoplasm” and “Breast Cancer”. However, GS related to telomere maintenance was found to be significantly downregulated in LCL-AT. DE-GS related to immunity were also particularly deregulated in LCL-AT, suggesting that LCL-AT may have immunological disorders. There was also a high enrichment of cytokines and interleukin GS in LCL-AT. Other DE-GS related to immunity included NK-cell cytotoxicity, abnormal innate immunity or abnormal level of class II molecules (Fig. 3).

### Analysis of the viral gene expression

**Fig. 4:**
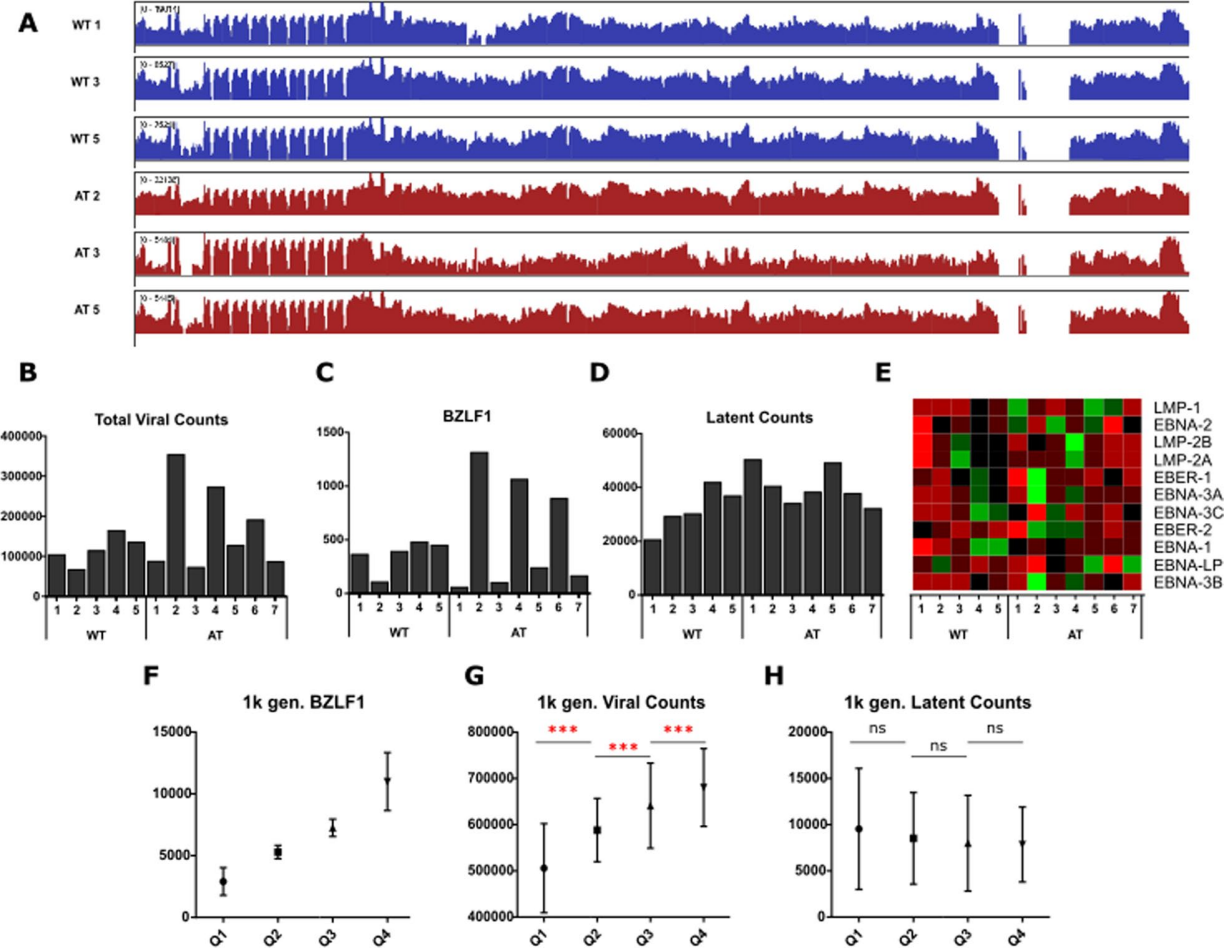
RNA seq results of the viral genome. **a** Whole genome view of RNA-seq coverage across the EBV genome on representative samples of LCL-WT and LCL-AT. The y-axis shows the number of reads mapping to each location of the EBV genome on a logarithmic scale. **b** Results of reads alignment on the viral genome. The number of normalized reads is indicated on the y-axis. **c** Normalized BZLF1 reads show the expression pattern as total normalized reads. **d** Normalized latent counts were extracted from total viral counts. **e** Heatmap displaying all latent genes in each LCL-WT and LCL-AT. Red represent downregulated genes and green represent upregulated genes. **f**–**h** Analysis of EBV read counts from the 1000 genome project (1 k gen.). **f** 464 EBV RNA-seq results were separated in 4 groups according to the expression of BZLF1 (quantile Q1–Q4). For each of these quantile, total viral counts (G) and latent counts (H) were determinated. ***pval < 0.001

The normalized viral counts were viewed using the IGV software (Fig. 4a). A zoom on an intergenic region showed no reads, supporting the absence of contaminating reads from cellular DNA (Additional file 7: Fig. S4). The AT2, AT4 and AT6 lines express more viral transcripts than the other lines (Fig. 4b). This could be explained by a difference in lytic cycle induction between all lines at the time of RNA extraction. Indeed, there is always 0.1–4% of cells undergoing lytic cycle [17]. EBV-infected cells in the latent phase express 9 latent genes and 2 non-coding EBERs, whereas during the lytic cycle the cells express around a hundred lytic genes under the control of BZLF1, the lytic cycle master-gene regulator [1]. Knowing that cells in latent cycle express little viral RNA, a small difference in the proportion of cells undergoing lytic cycle would be enough to drastically change the total number of viral transcripts expressed in each line. Indeed, we confirmed that the expression of BZFL1 follows the same pattern (Pearson correlation = 0.97) as the total viral counts (Fig. 4c).

To verify that the small proportion of cells undergoing lytic reactivation, do not significantly modify the overall latent gene expression, we downloaded 464 EBV standardized read counts from the EBV Portal platform [18], which gather EBV RNA-seq results of LCL-WT sequenced within the 1000 genome project. We established 4 quartiles according to BZLF1 expression, (Fig. 4f). Total and latent viral counts were calculated for each quartile. The results show that total viral counts depend on BZLF1 expression, with a significant increase in total viral counts between each quartile (Fig. 4g), but not latent viral counts (Fig. 4h). Total viral counts are relative to BZLF1 expression and have no correlation with latent counts. This allows us to analyze latent genes, regardless of lytic genes expression.

**Fig. 5:**
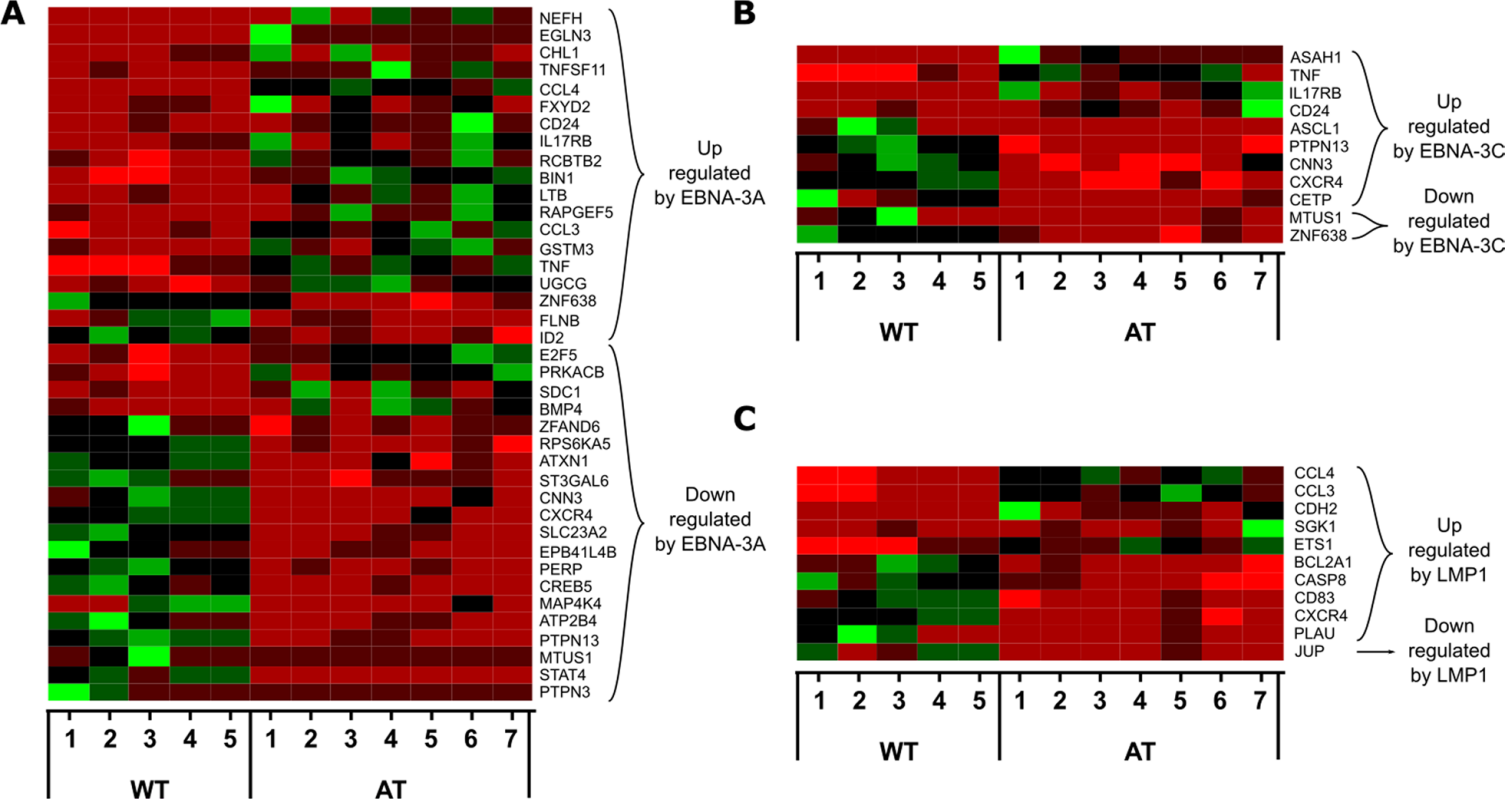
Heatmaps of EBV interactome. **a**–**c** Heatmaps showing all differentially expressed genes that were described, according to the Polygenicpathway EBV database, as regulated by: **a** EBNA-3A, **b** EBNA-3C, **c** LMP1

Differential analysis of latent genes did not show any DE genes between LCL-AT and LCL-WT (Fig. 4d, e) and all latent genes were expressed in LCL-WT and LCL-AT. We compared the cellular DE genes list with the non-redundant viral latent genes interactome available on the Polygenic pathways database. From this analysis we found 71 interactions representing 54 DE genes, of which 42 have an |log2FC|> 1. EBNA-3A appears to interact with 39 DE genes (Fig. 5a), EBNA-3C with 11 DE genes (Fig. 5b) and LMP1 with 11 DE genes (Fig. 5c). These data suggest that although the EBNA-3A, EBNA-3C and LMP1 transcripts were not DE, the activity of these genes may be disrupted in LCL-AT. Interestingly, most of the DE genes described as downregulated or upregulated by EBNA-3A were also downregulated or upregulated in LCL-AT respectively, indicating that EBNA-3A may be more active in LCL-AT than in LCL-WT.

## Discussion

AT patients have a high risk of developing lymphoid malignancies with a high rate of association with EBV. Mechanisms associated with this specific susceptibility may be due cellular immune deficiency in a number of AT patients, or to other specific immune dysfunctions that remain to be explored. We raised the hypothesis that ATM defect in EBV-infected cells could play a role per se in the control of EBV latency, favoring a latent program more prone to lymphomagenesis [19]. In the present study, we used strand-specific RNA seq strategy to profile the RNA expression landscape of ATM deficient LCL versus control, in order to assess the involvement of ATM in EBV latent cycle regulation.

Our data suggests a previously unsuspected ribosomal defect in LCL-AT. In addition, we found that LCL-AT display a distinct pattern of cancer associated gene expression, most notably by overexpressing certain oncogenes and downregulation of tumor suppressors, and also exhibit features of immune dysfunction. We also confirmed that latent gene expression can be studied regardless of lytic gene expression. Our data, based on the single technique of RNA-seq analysis, will require validation by additional studies.

### Transcription, translation and mitochondria

Our results suggest that LCL-AT may have a transcriptional and translational defect. Indeed LCL-AT express less 28S RNA and have a lower translational rate than LCL-WT, but transcription capacities did not differ significantly from LCL-WT. The possible transcriptional defect does not appear to affect EBV latency genes, as observed by the absence of DE latency genes between LCL-AT and LCL-WT. Housekeeping genes were not DE, suggesting that transcriptional alterations may affect specific genes such as ribosomal genes. Interestingly, the signaling pathways for transcription and translation were not found in the EGSEA results but stand out significantly with a lower log.fold.dir. This underscores the need for several complementary methods to study RNA-seq data.

The alteration of 28S RNA and translational rate in LCL-AT suggest that the pathophysiology of ataxia-telangiectasia may also include aspects of ribosomal disease. ATM participates in the modification of the nucleolus architecture in case of double-strand break within rDNA [20] and It has been suggested that ATM participates in basal nucleolar transcription [21]. Other immunodeficiencies with specific susceptibility to EBV—such as *CTPS1* deficiency—are characterized by altered nucleic acid metabolism leading to rapid T-cell exhaustion upon massive proliferation induced by EBV infection [22]. We hypothesize that the massive protein synthesis rate in cytotoxic T-cells during EBV-driven proliferative stress is inefficiently sustained in ataxia-telangiectasia, resulting in a defective control of EBV. Further studies are needed to address this hypothesis. Transcription of many mitochondrial genes were decreased in LCL-AT including several genes involved in the respiratory chain and in ribosomal protein synthesis. Inhibition of ATM leads to mitochondrial dysfunction and ROS production [23]. The latter could be involved in the increased incidence of cancers in patients with ataxia-telangiectasia by increasing genotoxic stress.

### Oncogenesis and immune dysfunction

The EGSEA results show enrichment in pro-tumorigenic GS particularly oncogenes, growth factors and downregulation of tumor suppressors in keeping with the increased cancer risk in AT [24]. Among the main oncogenes induced in LCL-AT, we find BCL11A (log2FC 4.20), a modulator of transcriptional repression frequently upregulated in B-cell malignancies [25, 26] or TCL1A (log2FC 3.41), a survival promoting factor strongly associated with Burkitt lymphoma and related to other malignancies [27, 28]. The main tumor suppressors downregulated in AT are PCDH10 (log2FC − 4.76), a protocadherin whose promoter is methylated in diffuse large B-cell lymphomas [29] or PTPN13 (log FC: − 2.84) an inhibitor of FAS-induced apoptosis associated with aggressive breast cancer [30].

Telomere maintenance pathway, including *TERT,* was downregulated in LCL-AT, (log2FC − 4.78). Inhibition of TERT in LCL decreases cell proliferation and induces apoptosis in an ATM dependent manner [31] as well as induces the EBV lytic cycle, which was not the case in LCL-AT (data not shown). LCL-AT may use an alternative lengthening of telomeres pathway [32]. Whether TERT related induction of the EBV lytic cycle is also ATM dependent should be further explored.

A modulation of innate immunity in LCL-AT is suggested by several DE-GS. The gene expression of HLA-C, a major NK cell inhibitory molecule, is upregulated in LCL-AT (log2FC 5.63). Similarly, CD200R1, CD276, SLAMF7, LILRB1 were overexpressed, suggesting that AT patients may have a disrupted NK cell function. On the other hand, LAIR1, another inhibitory molecule, was downregulated (log2FC − 4.99).

IL4 and IL10 were also upregulated in LCL-AT (log2FC 2.23 and 3.58, respectively). These two cytokines participate in the proliferation, plasma cell differentiation and antibody production of B lymphocytes [33, 34]. IL10 also inhibits CD8 cytotoxic T-cells [35]. The cGAS, STING (TMEM173) [36] and interferon β1 (IFNB1) transcripts were downregulated (log2FC − 0.70; − 2.39 and − 2.51 respectively), suggesting a possible defect in antiviral response in LCL-AT.

### EBV regulation

We found no significant difference in EBV latent gene expression between LCL-AT and LCL-WT. Several deregulated cellular genes in LCL-AT interact with EBNA-3A, EBNA-3C and LMP1 suggesting an overall differential impact on cellular homeostasis.

ATM participates in the regulation of EBV’s lytic cycle and is necessary for a proper viral replication in epithelial cells [37]. In LCL however, ATM inhibition through caffeine treatment [38] or the lack of ATM in our LCL-AT did not affect viral replication. On the other hand, LCL treated by the pan-PIKK inhibitor (to which the ATM kinase belongs) LY294002 were shown to inhibit viral replication. It is thus possible that another kinase compensates for ATM deficiency in LCL, to promote viral replication. ATR is a good candidate as it activates the same downstream targets as ATM.

However, the impact of ATM on the control of EBV latency may not be manifested in a highly artificial system such as LCL, but appear in the context of natural EBV-B cell infection.

## Conclusion

In summary, we show that LCL-AT display a gene expression pattern consistent with the observed increased incidence of EBV-related malignancies in patients with Ataxia-Telangiectasia. The dysregulated pathways uncovered by this approach need to be further explored to better understand the biological mechanisms involved in the regulation of EBV latency and lymphomagenesis. Elucidation of these pathways may contribute to the development of novel approaches to treat or prevent EBV associated lymphoproliferations in AT patients where conventional chemotherapy is very toxic because of the DDR defect, and also in the general population.

## Methods

### Cell lines and culture

LCL were generated according to standard protocols at the Genethon Laboratory and at the Imagine Institute Biological Resource Center. LCL were cultured in RPMI containing 10% FBS at 37 °C in a 5% CO_2_ incubator and passed every 3 days replacing half of the culture medium with fresh medium.

### RNA extraction and sequencing

LCL-WT and LCL-AT were harvested in an exponential growth phase. Total RNA was extracted using the RNeasy Plus Mini Kit (Qiagen). The concentration of total RNA was measured spectrometrically using Xpose (Trinean). The RNA integrity was analyzed by capillary electrophoresis using a Tape-Station (Agilent). The NUGEN Ovation Universal RNA-Seq system was used to construct the RNA-seq libraries from 100 ng of total RNA according to the manufacturer protocol. RNA sequencing was performed by the genomics platform of Imagine Institute on HiSeq 2500 (Illumina), by multiplexing 12 libraries per line to obtain a sequencing depth of 70 million pair-end reads per library, with a read length of approximately 130 nucleotides.

### Reads quantification and differential analysis

Salmon v0.8.2 [39] was used to pseudo-align raw RNA-seq reads to both human and viral genomes and get quantification estimates at the transcript level. The human reference genome GRCh38 was downloaded from the ENSEMBL website [40] (http://ftp.ensembl.org/pub/release-92/fasta/homo_sapiens/cdna), and the EBV genome B95-8/Raji from Flemington Lab (http://www.flemingtonlab.com/rnaseq.html). Differential analysis was performed using R software, version 3.5.0 [41] and the negative binomial generalized linear modelling implemented in DESeq2 package version 1.20.0 [42]. A Wald test was applied on viral transcripts to perform comparisons among conditions. *p* Values were corrected for multiple testing using Benjamini–Hochberg correction [43]. The BioMart R package [44] was used to annotate human differentially expressed genes.

### Genome visualization

Reads were mapped on the human genome (hg38) using STAR v2.5.0a [45] and the unmapped reads were aligned to the EBV genome using bowtie2 v2.3.4.2 [46]. The format conversions, sorting, and indexing intermediate operations on the data were performed using samtools v1.7 [47] and bedtools v2.26 [48]. The snapshots obtained in Fig. 4a and Additional file 7: Fig S4 were obtained with IGV v2.4.15 [49].

### Gene ontology and gene sets analysis

Differentially expressed genes were subjected to GO-Analysis, using the over-representation test within the PANTHER classification system [50] (version 13.1). Statistically over-represented GO-categories (FDR < 0.05) containing less than 25% of the input were selected for visualization. Functional analysis was carried out using the Ensemble of Gene Set Enrichment Analyses (EGSEA) R package (version 1.8.0). EGSEA uses 12 algorithms and combine the results by calculating a Wilkinson adjusted p-value. The EGSEA database consists of approximately 25.000 genes-sets classified into 16 collections from the MSigDB [51, 52], GeneSetDB [53] and KEGG [54] databases.

### Transcription and translation assay

Cells were incubated with 5-ethynyl uridine (EU) on 1 h or with O-Propargyl-puromycin (OPP) on 30 min. Transcription and translation rate were assessed using the Click-it assay Kit (Invitrogen), followed by flow-cytometer analysis. Statistical comparison between LCL-AT and LCL-WT were performed using a Mann–Whitney–Wilcoxon non-parametric test, using Prism (version 7.00, GraphPad Software).

### 1000 Genome project and EBV interactome data

EBV standardized read counts sequenced within the 1000 genome project were downloaded on the EBV Portal platform (https://ebv.wistar.upenn.edu) and the EBV interactome data from the Polygenic pathways website (http://www.polygenicpathways.co.uk/epsteinbarr.htm).

## Supplementary Information

**Additional file 1: Figure S1.** ATM expression and exploration of its function.

**Additional file 2: Table S1** List of 1899 differentially expressed genes.

**Additional file 3: Figure S2.** GO analysis of differentially expressed genes.

**Additional file 4: Table S2** List of Mitochondrial, RNA-related and ribosome-related differentially expressed genes.

**Additional file 5: Figure S3.** Exploration of rRNA content.

**Additional file 6: Table S3.** EGSEA results for differentially expressed genes.

**Additional file 7: Figure S4.** RNA-seq coverage across an intergenic region of EBV genome.

## Data Availability

All data generated or analyzed during the current study are included in this published article.

## Abbreviations

AT: ataxia-telangiectasia
|avg.logfc.dir|: average log2FC with genes regulated in the same direction as the GS
DDR: DNA damage response
DE: differentially expressed
EBV: Epstein–Barr virus
EGSEA: ensemble of gene set enrichment analyses
EU: 5-ethynyl uridine
GS: gene-set
LCL: lymphoblastoid cell line
|log2FC|: absolute Log2 fold-change
OPP: O-propargyl-puromycin
PANTHER: protein analysis through evolutionary relationships
PID: primary immune deficiencies
RIN: RNA integrity number
