# Supplementary material for: Gene expression analysis in EBV-infected ataxia-telangiectasia cell lines by RNA-sequencing reveals protein synthesis defect and immune abnormalities
Source: Orphanet J Rare Dis. 2021 Jun 28;16:288. doi: 10.1186/s13023-021-01904-3 (PMC8237493; doi:10.1186/s13023-021-01904-3)
Supplement: Supplementary file 1 — Additional file 1: Figure S1. ATM expression and exploration of its function. [file 13023_2021_1904_MOESM1_ESM.docx]

**Additional file 1: Figure S1**

To ensure the phenotype of LCL-WT and LCL-AT in our possession, the expression of ATM protein was tested by Western-Blot. Within LCL-WT, all lines express the ATM protein with a slight decrease in expression for the WT2 line compared to the others. Within LCL-AT, an absence of expression of ATM protein is observed, except for the AT4 and AT6 lines where ATM is expressed. ATM mutations during AT are in the majority of cases nonsense mutations leading to a complete absence of expression of the ATM protein. However, mutations leading to an expressed but non-functional protein have also been described. The AT4 and AT6 lines were therefore subjected to a functional test to verify the activity of ATM. In cas of double strand break, ATM phosphorylates H2AX at the gamma position, allowing it to recruit other repair proteins. Phosphorylation of the γH2AX protein indicates the presence of a functional ATM protein because no other kinase is able to phosphorylate it. The WT6 and WT7 lines were not used in the RNA-seq exploration.


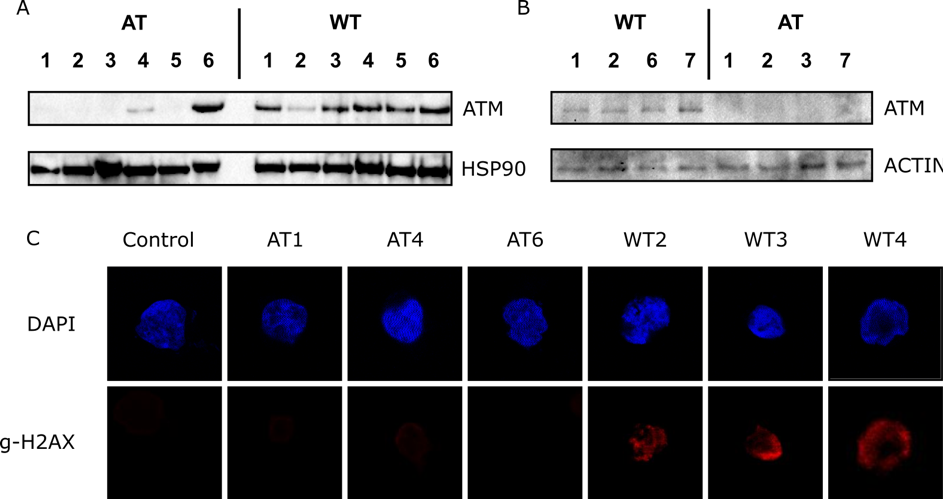


**Additional file 1: Figure S1: ATM expression and exploration of its function.** (A-B) ATM expression was assessed by Western Blot, using HSP90 (A) or actin (B) as a load control. Due to the number of samples, analysis could not be performed on a single blot. (C) Immunofluorescence of the γH2AX protein after irradiation of the cell-lines with 5Gy (bottom), the nuclei of the different cells were visualized by DAPI labeling (top). Control corresponds to the use of secondary antibody alone on an irradiated LCL-WT line.
