# Supplementary material for: Gene expression analysis in EBV-infected ataxia-telangiectasia cell lines by RNA-sequencing reveals protein synthesis defect and immune abnormalities
Source: Orphanet J Rare Dis. 2021 Jun 28;16:288. doi: 10.1186/s13023-021-01904-3 (PMC8237493; doi:10.1186/s13023-021-01904-3)
Supplement: Supplementary file 3 — Additional file 3: Figure S2. GO analysis of differentially expressed genes. [file 13023_2021_1904_MOESM3_ESM.docx]

**Additional file 3: figure S2**


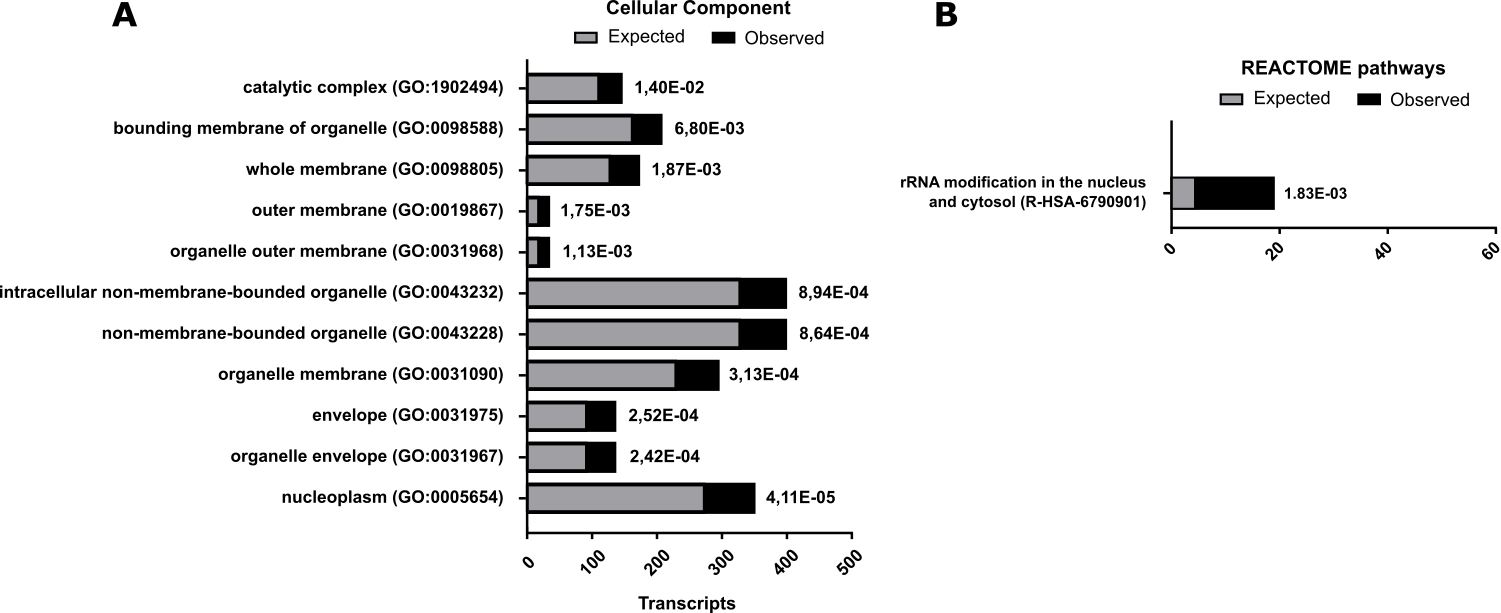


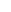


**Additional file 3: Figure S2: GO analysis of differentially expressed genes.** The PANTHER statistical over-representation test tool was used to determine over-representation of defined functional classes for the 1899 differentially expressed genes. These 11 GO categories corresponding to cellular component were judged as too general and non-informative, and were eliminated for better clarity of Figure 2C.
