# Supplementary material for: Gene expression analysis in EBV-infected ataxia-telangiectasia cell lines by RNA-sequencing reveals protein synthesis defect and immune abnormalities
Source: Orphanet J Rare Dis. 2021 Jun 28;16:288. doi: 10.1186/s13023-021-01904-3 (PMC8237493; doi:10.1186/s13023-021-01904-3)
Supplement: Supplementary file 5 — Additional file 5: Figure S3. Exploration of rRNA content. [file 13023_2021_1904_MOESM5_ESM.docx]

**Additional file 5: figure S3**


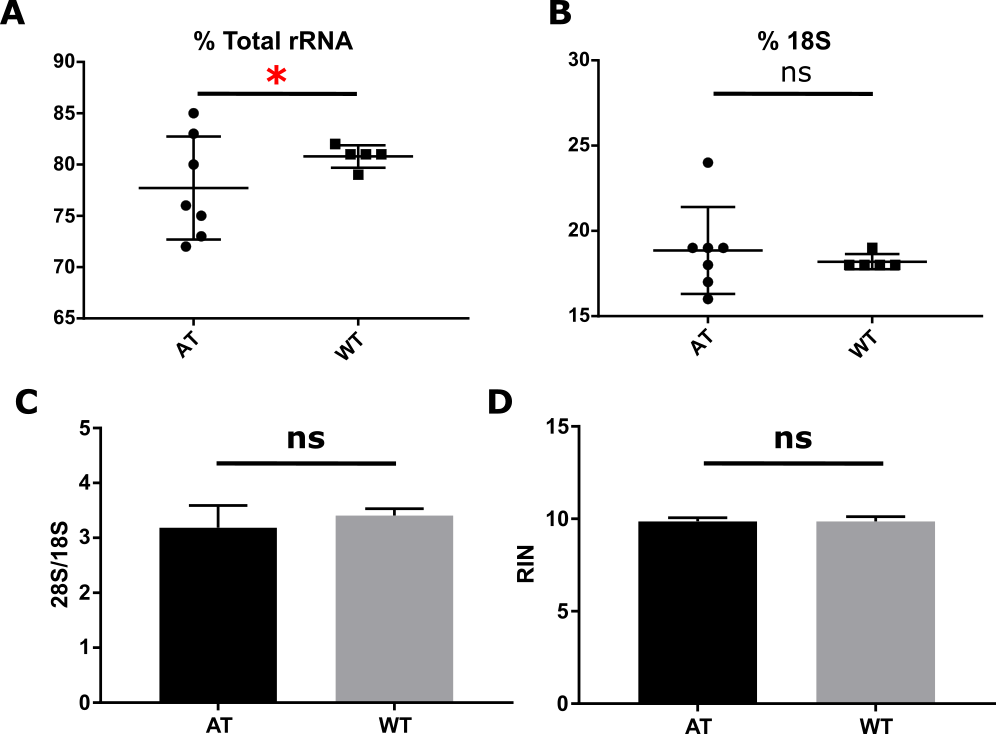


**Additional file 5: Figure S3: Exploration of rRNA content.** (A-B) Expression of rRNA in LCL-AT and LCL-WT shown as a percentage of total RNA expression: (A) Total rRNA, (B) 18S rRNA. (C-D) The quality control of RNA degradation was assessed for LCL-AT and LCL-WT using two methods: (C) calculating the 28S/18S ratio, (D) determining the RNA integrity number (RIN).
