# Supplementary material for: Gene expression analysis in EBV-infected ataxia-telangiectasia cell lines by RNA-sequencing reveals protein synthesis defect and immune abnormalities
Source: Orphanet J Rare Dis. 2021 Jun 28;16:288. doi: 10.1186/s13023-021-01904-3 (PMC8237493; doi:10.1186/s13023-021-01904-3)
Supplement: Supplementary file 7 — Additional file 7: Figure S4. RNA-seq coverage across an intergenic region of EBV genome. [file 13023_2021_1904_MOESM7_ESM.docx]

**Additional file 7: Figure S4**


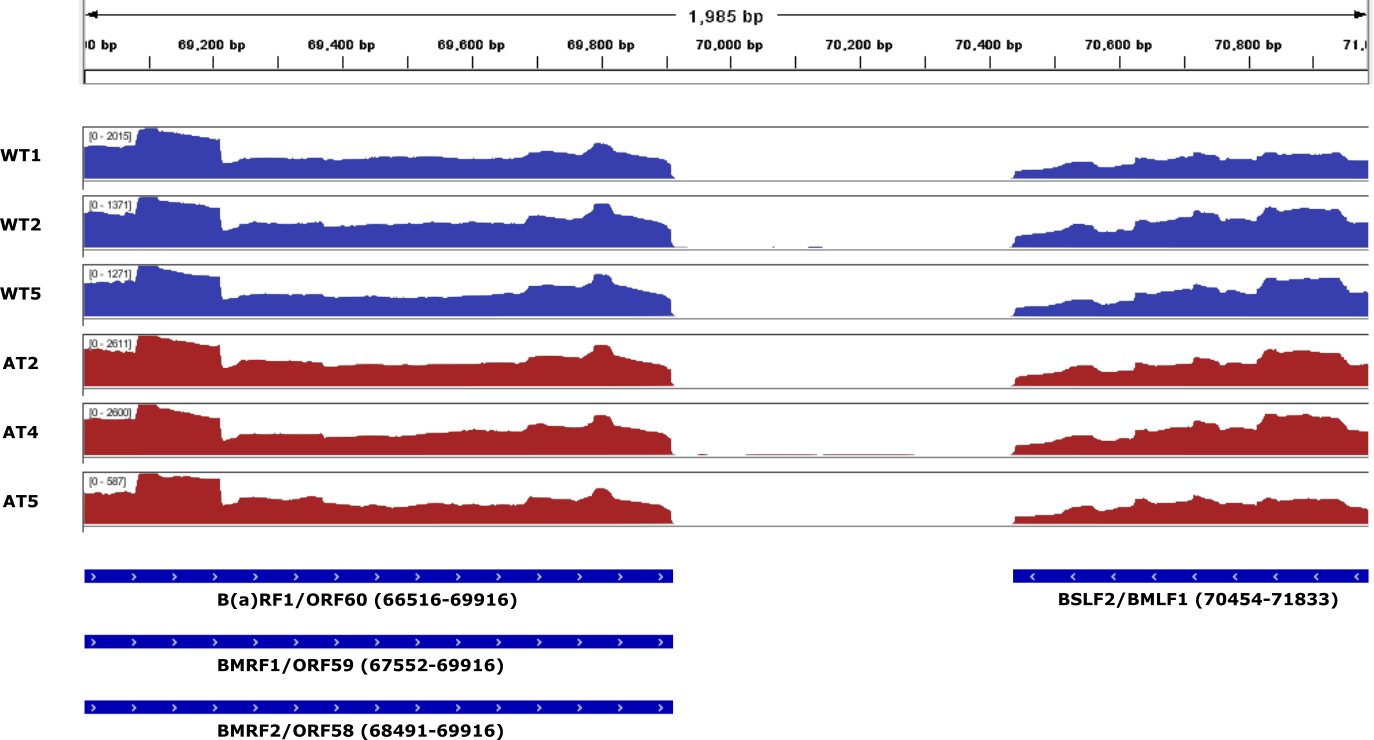


**Additional file 7: Figure S4: RNA-seq coverage across an intergenic region of EBV genome.** Zoom on a 1985 bp region of EBV genome showing the intergenic region between the end of the EBV genes B(a)RF1/ORF60, BMRF1/ORF59, BMRF2/ORF58 on one side and BZLF2/BMLF1 on the other side. The number in brackets represents the start and the end position of the unique exon of each of these genes, (reference EBV genome V01555.2). The y-axis of each graph shows the number of reads mapping to each location of the EBV genome on a logarithmic scale.
